# Phylogeny and diversification of genus *Sanicula* L. (Apiaceae): novel insights from plastid phylogenomic analyses

**DOI:** 10.1186/s12870-024-04750-0

**Published:** 2024-01-24

**Authors:** Bo-Ni Song, Chang-Kun Liu, An-Qi Zhao, Rong-Ming Tian, Deng-Feng Xie, Yu-Lin Xiao, Huai Chen, Song-Dong Zhou, Xing-Jin He

**Affiliations:** 1 Key Laboratory of Bio-Resources and Eco-Environment of Ministry of Education, College of Life Sciences, Sichuan University, Chengdu, 610065 China; 2 CAS Key Laboratory of Mountain Ecological Restoration and Bioresource Utilization & Ecological Restoration and Biodiversity Conservation Key Laboratory of Sichuan Province, Chengdu Institute of Biology, Chinese Academy of Sciences, Chengdu, China

**Keywords:** Apiaceae, *Sanicula*, Plastome, DNA barcodes, Phylogeny, Divergence time

## Abstract

**Background:**

The genus *Sanicula* L. is a unique perennial herb that holds important medicinal values. Although the previous studies on *Sanicula* provided us with a good research basis, its taxonomic system and interspecific relationships have not been satisfactorily resolved, especially for those endemic to China. Moreover, the evolutionary history of this genus also remains inadequately understood. The plastid genomes possessing highly conserved structure and limited evolutionary rate have proved to be an effective tool for studying plant phylogeny and evolution.

**Results:**

In the current study, we newly sequenced and assembled fifteen *Sanicula* complete plastomes. Combined with two previously reported plastomes, we performed comprehensively plastid phylogenomics analyses to gain novel insights into the evolutionary history of this genus. The comparative results indicated that the seventeen plastomes exhibited a high degree of conservation and similarity in terms of their structure, size, GC content, gene order, IR borders, codon bias patterns and SSRs profiles. Such as all of them displayed a typical quadripartite structure, including a large single copy region (LSC: 85,074–86,197 bp), a small single copy region (SSC: 17,047–17,132 bp) separated by a pair of inverted repeat regions (IRs: 26,176–26,334 bp). And the seventeen plastomes had similar IR boundaries and the adjacent genes were identical. The *rps*19 gene was located at the junction of the LSC/IRa, the IRa/SSC junction region was located between the *trn*N gene and *ndh*F gene, the *ycf*1 gene appeared in the SSC/IRb junction and the IRb/LSC boundary was located between *rpl*12 gene and *trn*H gene. Twelve specific mutation hotspots (*atp*F, *cem*A, *acc*D, *rpl*22, *rbc*L, *mat*K, *ycf*1, *trn*H*-psb*A, *ycf*4*-cem*A, *rbc*L*-acc*D, *trn*E*-trn*T and *trn*G*-trn*R) were identified that can serve as potential DNA barcodes for species identification within the genus *Sanicula*. Furthermore, the plastomes data and Internal Transcribed Spacer (ITS) sequences were performed to reconstruct the phylogeny of *Sanicula.* Although the tree topologies of them were incongruent, both provided strong evidence supporting the monophyly of Saniculoideae and Apioideae. In addition, the sister groups between Saniculoideae and Apioideae were strongly suggested. The *Sanicula* species involved in this study were clustered into a clade, and the *Eryngium* species were also clustered together. However, it was clearly observed that the sections of *Sanicula* involved in the current study were not respectively recovered as monophyletic group. Molecular dating analysis explored that the origin of this genus was occurred during the late Eocene period, approximately 37.84 Ma (95% HPD: 20.33–52.21 Ma) years ago and the diversification of the genus was occurred in early Miocene 18.38 Ma (95% HPD: 10.68–25.28 Ma).

**Conclusion:**

The plastome-based tree and ITS-based tree generated incongruences, which may be attributed to the event of hybridization/introgression, incomplete lineage sorting (ILS) and chloroplast capture. Our study highlighted the power of plastome data to significantly improve the phylogenetic supports and resolutions, and to efficiently explore the evolutionary history of this genus. Molecular dating analysis explored that the diversification of the genus occurred in the early Miocene, which was largely influenced by the prevalence of the East Asian monsoon and the uplift of the Hengduan Mountains (HDM). In summary, our study provides novel insights into the plastome evolution, phylogenetic relationships, taxonomic framework and evolution of genus *Sanicula*.

**Supplementary Information:**

The online version contains supplementary material available at 10.1186/s12870-024-04750-0.

## Background

*Sanicula* L. is a unique genus of perennial woodland herbs belonging to Apiaceae subfamily Saniculoideae [1]. The genus consists of about forty species worldwide that are widely distributed in Asia, North America, Europe and Africa [1–4], with two distribution centers: China and North America. The relatively widespread distribution of this genus is mainly due to the hooked prickles on the fruit that can stick to animals, which facilitate the spread of these species to further areas [5].

 The genus, known as black snakeroot or sanicle, was established by Linnaeus in 1753 with the type species *S. europaea* L [1, 6]. The main morphological features of its members are characterized by spiny or bristly simple leaves, simple (rarely compound) umbels or heads, showy bracts, conspicuously prominent and persistent calyx, two persistent styles and fruits covered with scales, bristles, or hooked prickles, which can easily distinguish them from other genera of the Apiaceae [1, 7]. However, the genus always exhibits great variability in habit, foliage, flowers and fruits [8], thus some researchers were prone to divide the genus into more smaller classification units. For example, De Candolle separated the genus into two sections: *Sanicla* DC. and *Sanicoria* DC. [9, 10] relied on leaf and fruit morphology. However, Drude divided the genus into three subgenera (*Sanicula* L., *Sanicoria* DC. and *Erythrosana* Drude) and seven sections (*Rosiflorae* Drude, *Flaviflorae* Drude, *Orthospermae* Drude, *Camplospermae* Drude, *Pinnatae* Drude, *Tuberculatae* Drude and *Tuberosae* Drude) based on the petal color, seed surface shape and fruit prickles [11]. On the basis of Drude, Wolff divided the genus into ten sections [12]. Later, Shan and Constance proposed a completely revised treatment for this genus, who reduced the four sections of Wolff’s classification to the synonymy taxonomy in sect. *Sanicoria* DC and formed five sections (*Tuberculatae* Drude, *Pseudopetagnia* H.wolff, *Sanicula* DC., *Sandwicenses* Shan and Constance, *Sanicoria* DC.) based on differences in habit, flower, fruit and distribution [8]. Therefore, it can be seen that the classification system of genus *Sanicula* has long been disputed. In addition, species delimitation of this genus was also blurred, which was also mainly caused by the high variability of morphological characteristics. For example, *S. orthacantha* var. *stolonifera* Shan & S.L.Liou has long rhizomes with distinct nodes, so it was considered to be a variety of *S. orthacantha* S. Moore [13]. Li et al. observed that *S. pengshuiensis* M. L. Sheh & Z. Y. Liu was morphologically similar to *S. lamelligera* Wolff ex Kretsch, and thus treated it as a synonymy of the latter [14]. From previous studies, it can be seen that the traditional methods of distinguishing these species were mainly focused on their morphological features, whereas many species of the genus have always shown great morphological instability, which have lead to extreme difficulties in species classification and delimitation [8–12]. Therefore, more evidence are needed to re-evaluate the taxonomic system of the genus.

A robust phylogenetic framework can provide strong evidence for the taxonomy of the genus *Sanicula*. Hence, some molecular markers have been applied to the phylogenetic studies of this genus, including single or multiple-locus DNA sequence data (for example ITS sequences, plastid DNA *rpl*16, *rps*16 intron and *rpoC*1 intron, *trrn*Q*-trn* K 5’-exon). Among these studies, Vargas P et al. observed that the sect. *Sanicoria* was not a monophyletic group and suggested that the circumscription of sect. *Sanicoria* should be enlarged to include the species of sect. *Sandwicenses* [5]. Valiejo et al. subsequently elucidated the relationships among the main genera of Saniculoidea based on nrITS sequences and found that sec. *Pseudopetagnia* and sect. *Sanicula* were also not monophyletic group [15]. In addition, there was much controversy about the interspecific relationships of the genus. For example, Vargas P et al. thought *S. orthacantha* was more closely related to *S. lamelligera* [5], while Yang et al. considered *S. orthacantha* had affinity to *S. chinese* [16]. However, these phylogenetic trees generated from DNA fragments had weak supports and low resolutions, and failed to provide sufficient information to support the improvement of taxonomy for *Sanicula* [5, 7, 17–19]. The sectional taxonomy system and the interspecific relationships of genus *Sanicula* are facing a severe challenge. Therefore, additional molecular resources are urgently needed to reconstruct a strongly robust phylogeny of genus *Sanicula* and re-evaluate the sectional taxonomy and interspecific relationships of the genus.

Besides, the evolutionary history of *Sanicula* is still poorly understood. Kadereit et al. proposed that the genus originated in the Miocene period of the Tertiary (15.5–6.0 Ma) based on the ITS sequences and plastid fragment *rps*16, combined with molecular clock [19]. Wen et al. indicated the origin of the genus was occurred at 16.54 Ma (95% HPD: 8.06–27.67 Ma) referred from 79 CDS data [20]. Vargas P et al. estimated the divergence time of sect. *Sanicoria* and sect. *Sandwicenses* relied on nrDNA sequences [5]. Although previous studies have laid the foundation for exploring the evolutionary history of the genus, the results obtained may be unreliable due to limited sampling and DNA fragments resulting in low supports and resolutions of the phylogenetic trees. Thus, a reliable phylogenetic framework and time tree are urgently needed to further investigate the evolutionary history of the genus based on expanded sampling.

Additionally, many species of *Sanicula* are valuable traditional herbs [21]. For example, *S. lamelligera*, known as “Fei Jingcao”, is a well-known traditional Chinese medicine that is widely used to treat cold, cough, tracheitis, bruises and amenorrhea [13, 22]. Other species such as *S. orthacantha* S. Moore., *S. orthacantha* var. *brevispina* de Boiss., *S. caerulescens* Franch., *S. chinensis* Bunge., *S. elata* Hamilt., and *S. astrantiifolia* Wolff ex Kretsch. are also Chinese medicinal herbs with the effects of resolving phlegm and cough, activating blood circulation and removing blood stasis [13]. Nevertheless, misuse of species names occurred frequently due to the abundant morphological variations within species, such as *S. chinensis* and *S. orthacantha*, as well as *S. caerulescens* and *S. lamelligera* [23], which made it difficult to identify species accurately. Hence, it is necessary to develop more efficient and specific DNA barcodes for *Sanicula* species authentication to ensure medicinal quality.

Plastid is a crucial and special multifunctional organelle involved in processes such as photosynthesis, carbon fixation and numerous major biological metabolic processes [24, 25]. The plastid genome (plastome) of flower plants ranges in size from 120 kb to 170 kb and displays a typical quadripartite structure, comprising a large single copy (LSC: 82–90 kb), a small single copy (SSC: 15–20 kb) and a pair of inverted repeats (IRa & IRb: 22–25 kb) [26]. It always encodes 110–130 unique genes, including about 80 protein-coding genes (PCGs), 30 transfer RNA (tRNAs) genes and four ribosomal RNA (rRNAs) genes [27]. The plastome is highly conserved in terms of its gene structure, sequence, content, order compared to nuclear and mitochondrial genomes [28, 29]. Moreover, the plastome is uniparental inheritance and possesses highly variable sites [30]. For these obvious advantages, the plastome has become a powerful tool to generate highly supported and resolved phylogenies and to explore more efficient specific DNA barcodes [25, 27, 28, 31–33]. In recent years, with the rapid development of next-generation sequencing technology and bioinformatics technology, a large number of sequence data of plastomes has become more accessible at a much lower cost [34], and it has been extensively and successfully applied to solve the plants phylogenies, especially for those taxonomically difficult taxa within the family Apiaceae [30–32, 35–40].

Plastomes also opened a promising window for disclosure of the genus *Sanicula*. Currently, although there were related studies on the plastomes of genus *Sanicula*, all of them only reported the *Sanicula* plastome structure or conducted a simple analyses, which failed to address the phylogenetic relationships of the genus [16, 41–43]. In the current study, we obtained seventeen plastomes of *Sanicula* species (fifteen newly sequenced and two previously reported) and performed comprehensively plastid phylogenomics analyses of this taxonomically difficult genus. Since the plastid genome is maternally inherited, it does not fully reveal the evolutionary history of the taxon, while ITS sequence is biparentally inherited and represents another different genetic pattern [30]. Therefore, to obtain a comprehensive understanding of phylogenetic relationships, we also used ITS sequence to construct the phylogenetic tree. Our major aims were to: (1) characterize the plastomes of *Sanicula* plants and select highly variable hotspot regions as candidate DNA barcodes for species authentication of *Sanicula*; (2) evaluate the potential of plastome for resolving the phylogeny of genus *Sanicula* and re-evaluate the section-level classification system and interspecific relationships; (3) investigate the evolutionary history of the genus.

## Results

### Basic characteristics of *Sanicula* plastomes

**Fig. 1:**
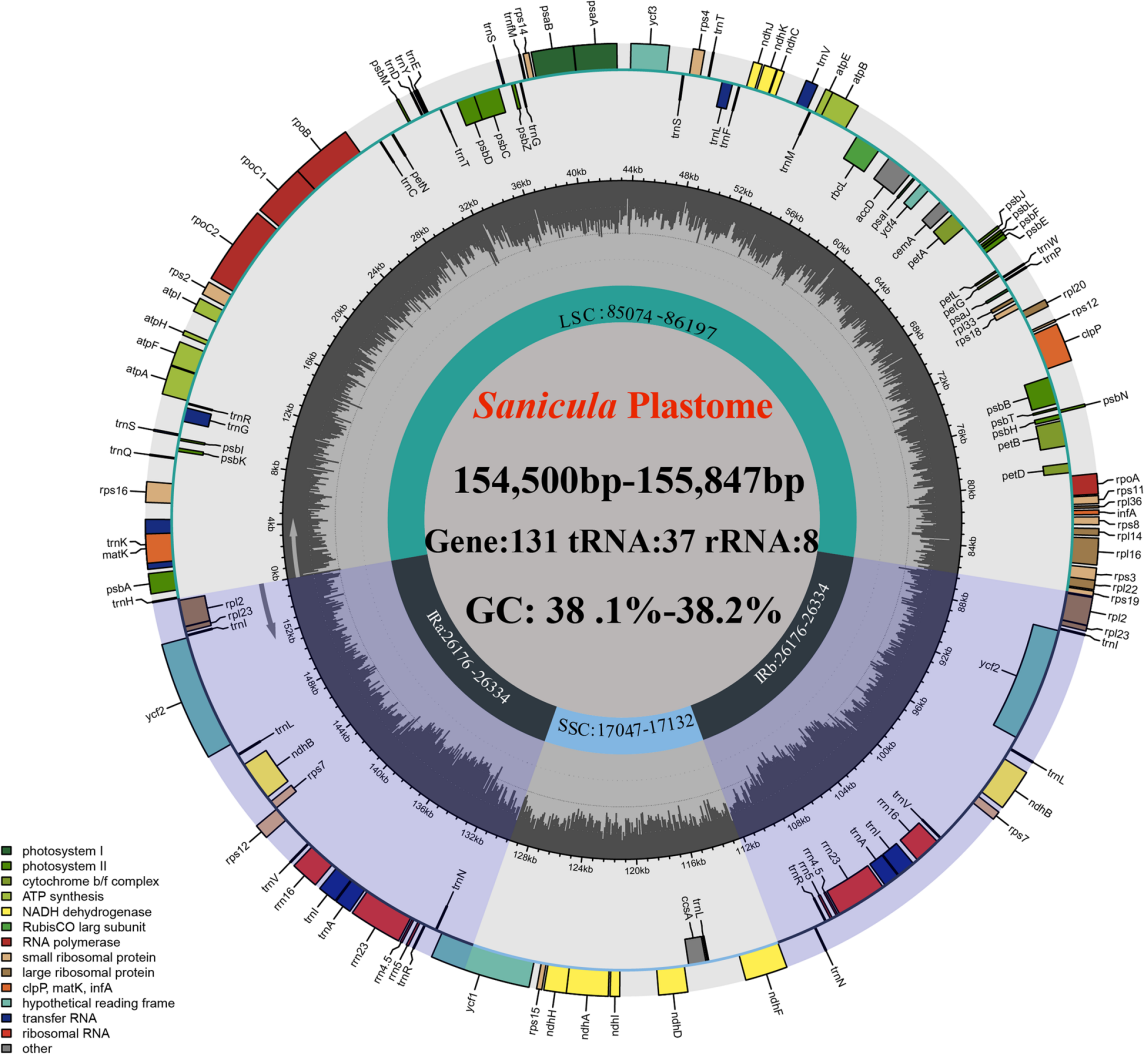
Whole plastome maps of seventeen *Sanicula* species. Genes showed outside of outward layer circle are transcribed clockwise, while those insides are transcribed counterclockwise. The genes belonging to different functional groups are color-coded. The pale gray area of the inner circle denotes the GC content of plastome

The complete plastome size of the seventeen *Sanicula* taxa ranged from 154,500 bp (*S. odorata* (Raf.) Pryer & Phillippe.) to 155,847 bp (*S. lamelligera*) (Table 1). All of them exhibited a typical quadripartite structure, including a large single copy region (LSC: 85,074–86,197 bp), a small single copy region (SSC: 17,047–17,132 bp) separated by a pair of inverted repeat regions (IRs: 26,176–26,334 bp) (Fig. 1; Table 1). The total GC content was account for 38.20%, except for *S. astrantiifolia* (38.10%); the IR regions had the highest GC content (42.9% – 43.0%) and the SSC region had the lowest GC content (32.4%– 32.6%). Four rRNAs (*rrn*16, *rrn*23, *rrn*4.5 and *rrn*5) genes possessed the same GC content (55.3%) among seventeen plastomes. All platomes encoded 113 unique genes, including 79 PCGs, 30 tRNA genes and 4 rRNA genes (Table 1) and these unique genes had four categories: Self-replication, Genes for photosynthesis, Other genes and Genes of unknown function. Among them, 46 coding genes were related to photosynthesis, which were divided into six group: Subunits of photosystem I, Subunits of photosystem II, Subunits of cytochrome, Subunits of ATP synthase, Large subunit of Rubisco and Subunits of NADH dehydrogenase. Seventeen genes contained intron and fourteen of them (*ndh*B, *trn*A, *ndh*A, *trn*I, *rpl*2, *rpl1*6, *pet*B, *trn*V, *trn*L, *rpo*C1, *atp*F, *trn*G, *rps*16 and *trn*K) harbored one intron and three of them (*clp*P, *rps*12 and *ycf*3) harbored two introns (Table S1).

**Table 1:** Comparison of plastome features among seventeen *Sanicula* plants

Taxon	Length (bp)				GC contents (%)				Number of genes (unique)			
	Genome	LSC	SSC	IR	Total	LSC	SSC	IR	Total	PCGs	rRNA	tRNA
*S. astrantiifolia*	155,748	86,140	17,096	26,256	38.10	36.4	32.5	42.9	113	79	4	30
*S. chinensis*	155,395	85,677	17,072	26,322	38.20	36.5	32.6	42.9	113	79	4	30
*S. caerulescens*	155,768	86,174	17,118	26,238	38.20	36.4	32.6	42.9	113	79	4	30
*S. elongata*	155,634	86,051	17,105	26,239	38.20	36.4	32.5	42.9	113	79	4	30
*S. giraldii*	155,609	85,857	17,086	26,333	38.20	36.5	32.5	42.9	113	79	4	30
*S. hacquetioides*	155,494	85,771	17,075	26,324	38.20	36.5	32.5	42.9	113	79	4	30
*S. lamelligera*	155,847	86,197	17,132	26,259	38.20	36.4	32.5	42.9	113	79	4	30
*S. orthacantha*	155,749	86,166	17,103	26,240	38.20	36.4	32.6	42.9	113	79	4	30
*S. orthacantha* var. *stolonifera*	155,396	85,818	17,098	26,240	38.20	36.4	32.5	42.9	113	79	4	30
*S. oviformis*	155,645	86,071	17,098	26,238	38.20	36.4	32.6	42.9	113	79	4	30
*S. pengshuiensis*	155,765	86,189	17,102	26,237	38.20	36.4	32.6	42.9	113	79	4	30
*S. rubriflora*	155,657	85,982	17,057	26,308	38.20	36.4	32.5	42.9	113	79	4	30
*S. rugulosa*	155,527	85,780	17,079	26,334	38.20	36.5	32.5	42.9	113	79	4	30
*S. serrata*	155,599	86,046	17,097	26,228	38.20	36.5	32.4	42.9	113	79	4	30
*S. tienmuensis*	155,717	86,075	17,106	26,268	38.20	36.4	32.5	42.9	113	79	4	30
*S. odorata*	154,500	85,074	17,074	26,176	38.20	36.5	32.5	42.9	113	79	4	30
*S. flavovirens*	155,335	85,682	17,047	26,217	38.20	36.4	32.5	43.0	113	79	4	30

### Codon usage bias and relative synonymous codon usage (RSCU) analysis

**Fig. 2:**
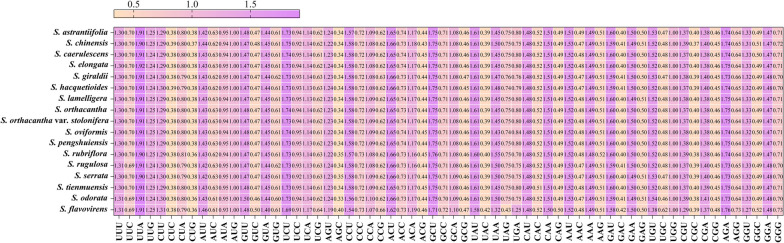
The RSCU values of 53 protein coding regions for seventeen *Sanicula* plastomes. The purple represents higher RSCU values, while the yellow indicates lower RSCU values

In order to analyze the codon usage of *Sanicula* plastomes, we screened the CDSs with the length more than 300 bp and finally attained 53 CDSs. We identified 21,162–21,202 codons in these seventeen plastomes, which have similar codon usage bias. The Leu had the highest number of codons (2,210–2,240) and it was encoded by UUA, UUG, CUU, CUC, CUA and CUG. Ser was the second most abundant amino and it was encoded by AUU, AUC and AUA, while the Cys had the least codons (217–265) with UGU and UGC encoded except for TER (Table S2). Among these 64 codons, the codon AUU occurred the most and the frequency was reached to 850–874, while UGC was the least (51–82 occurrences) except three terminator codons (Fig. 2, Table S2). The most frequent terminator codons was UAA with 29–52 occurrences, while the other two were UAG (15–17) and UGA (14–49), respectively. The RSCU values of the 64 codons unchanged significantly and showed similar codon preferences with values ranging from 0.34 to 1.91 (Fig. 2). Notably, the RSCU values for thirty codons was greater than 1.00 and six codons (UUA, UCU, ACU, GCU, UAU and AGA) were regarded as “overrepresented” codons with RSCU values greater than 1.6 except for GAU. The remainging 32 codons had RSCU values less than 1, and fifteen of them (CUC, CUG, GUC, AGC, ACG, GCG, UAC, CAG, AAC, GAC, GAG, UGC, UGC, CGG and GGC) were considered to be “underrepresented” codons with RSCU values less than 0.6. Two codons (GCU and GUC) displayed no preferences (RSCU = 1). All codons with RSCU > 1.00 ended with an A or a U, except for UUG codon (Fig. 2).

### Repeat sequences and simple sequence repeats (SSRs) analysis

**Fig. 3:**
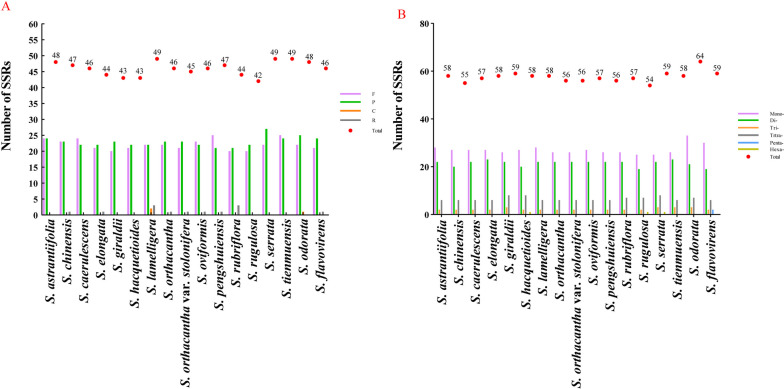
Analyses of repeats in the seventeen *Sanicula* plastomes. **A** Total number of four repeat types; (**B**) Total number of SSRs.

In this study, we detected 782 repeat sequences in seventeen plastomes and classified them into four types: forward repeats, palindromic repeats, reverse repeats, and complementary repeats. Among them, the most frequent repeats was the palindromic repeats (390), compared to other three types of repeats: forward repeats (376), reverse repeats (13) and complementary repeats (3) (Fig. 3A, Table S3). The species – *S. rugulos*a Diels. – had the least repeats (42), while three species (*S. lamelligera*, *S. serrata* H. Wolff., *S. tienmuensis* Shan & Constance.) had the most repeats (49). Forward and palindromic repeats were present in seventeen plastomes, and the complementary repeats only occurred in *S. lamelligera* and *S. odorata.* Nine *Sanicula* plastomes had reverse repeats. (Fig. 3A, Table S3).

In addition, we identified 979 SSRs in seventeen plastomes (Table S3). Among these SSRs, the mono-repeats were the most abundant (460, 46.99%), followed by di-repeats 365 (37.28%), tetra-repeats 111 (11.34%), tri-repeats 38 (3.88%), hexanucleotide repeats 3 (0.31%) and penta-repeatswere 2 (0.2%). All plastomes had the mono-repeats, di-repeats, tri-repeats and tetra-repeats (Fig. 3B). The penta-repeats and hexanucleotides were very rare, with penta-repeats (AAAAT/ATTTT and ACTAT/AGTAT) appearing only in *S. flavovirens* Z.H.Chen, D.D. Ma et W. Y. Xie. and hexanucleotide (ACATAT/ATATGT) occurring only in *S. hacquetioides* Franch., *S. rugulosa* and *S. serrata*. In the mono-repeats, A/T motifs were more abundant (24–32) than G/C motif (0–2). Likewise, AT sequences were also particularly rich in di-repeats and tri-repeats (Fig. 3B, Table S3).

#### Comparison of plastome analysis

**Fig. 4:**
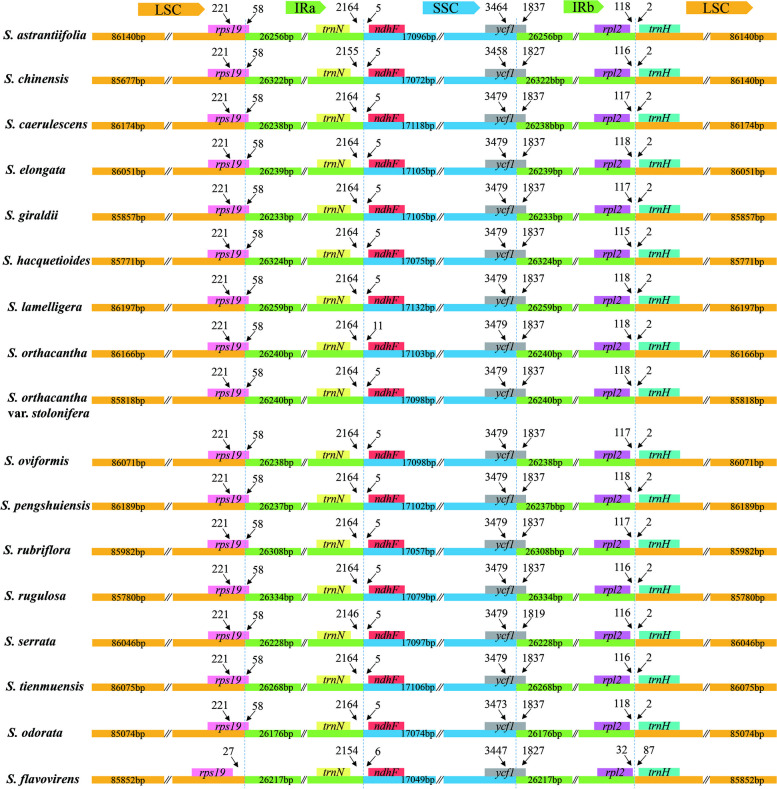
Comparison of IR borders among the seventeen *Sanicula* plastomes. Different boxes for genes represent the gene position

We examined the borders between inverted repeat and single-copy (IR/SC) among seventeen *Sanicula* plastomes.The results indicated that these *Sanicula* species had similar IR boundaries and adjacent genes were identical (Fig. 4). The *rps*19 gene was located at the junction of the LSC/IRa, and it had the same length of 221 bp in the LSC region and 58 bp in the IRa region in sixteen plastomes, but in *S. flavovirens*, the entire *rps*19 gene was located in the LSC region. The IRa/SSC junction region was located between the *trn*N gene and *ndh*F gene. The *trn*N and *ndh*F genes were 2,146–2,164 bp and 5–11 bp away from the IRa/LSC borders. The *ycf*1 genes, crossing the SSC/IRb borders, were located at the SSC and IRb regions with 3,447–3,479 bp and 1,819–1,837 bp. The IRb/LSC boundary was located between *rpl*2 gene and *trn*H gene. In these seventeen plastomes (except *S. flavovirens*), the *rpl*2 gene was 115–118 bp away from the IRb/LSC borders and the *trn*H gene was 2 bp away from the IRb/LSC borders, whereas in *S. flavovirens*, the *rpl*2 gene was only 32 bp away from the LSC region, and the *trn*H gene was 87 bp away from the IRb region (Fig. 4). Thus, these findings indicated that seventeen *Sanicula* plastomes were highly conservative.

**Fig. 5:**
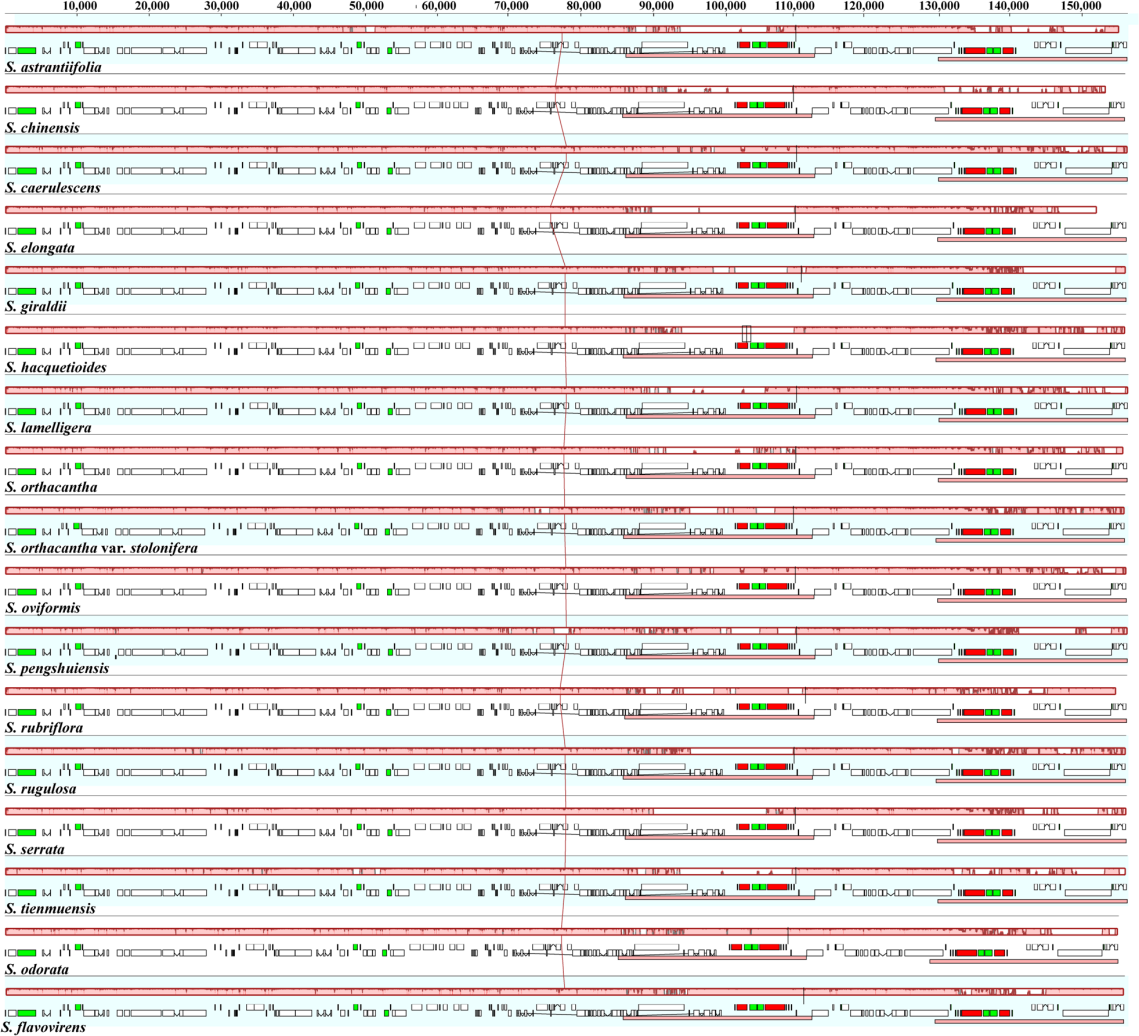
Mauve alignment of seventeen *Sanicula* plastomes. Local collinear blocks within each alignment are represented by blocks of the same color connected with lines

**Fig. 6:**
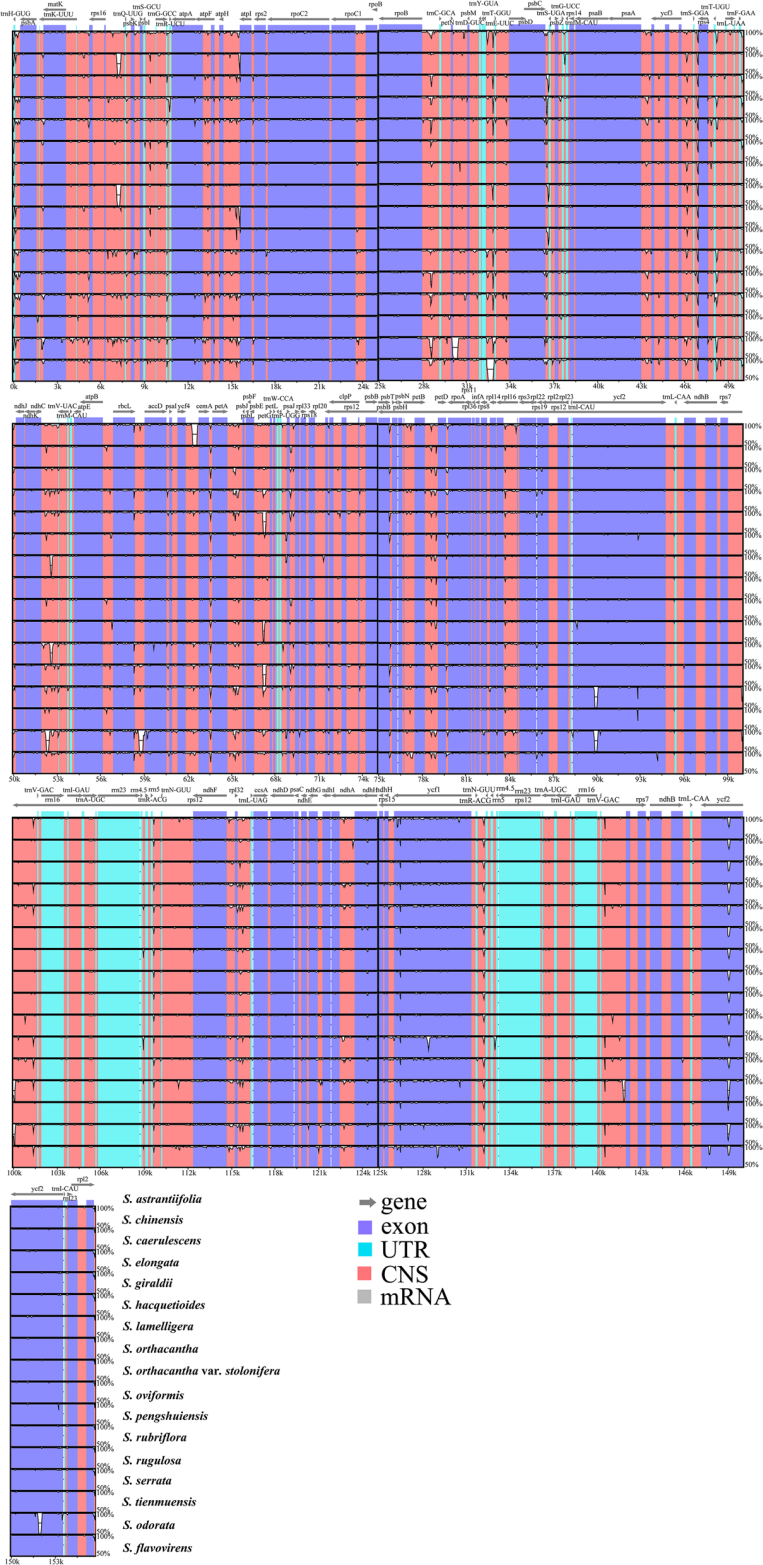
mVISTA alignment for seventeen *Sanicula* plastomes with *S. astrantiifolia* as the reference

The Mauve result revealed that gene arrangement of the seventeen plastomes was highly conserved (Fig. 5). The result performed by the mVISTA program illustrated that the plastomes of seventeen taxa were also highly conserved (Fig. 6). In addition, the mVISTA result found that the matrix contained 2472 variable sites (1.56%) and 1021 informative sites (0.65%) within the 15,8067 alignment positions. As a result, the whole plastomes sequence exhibited high similarity and no significant divergence was observed.

### Mutation hotspots in genus *Sanicula*

**Fig. 7:**
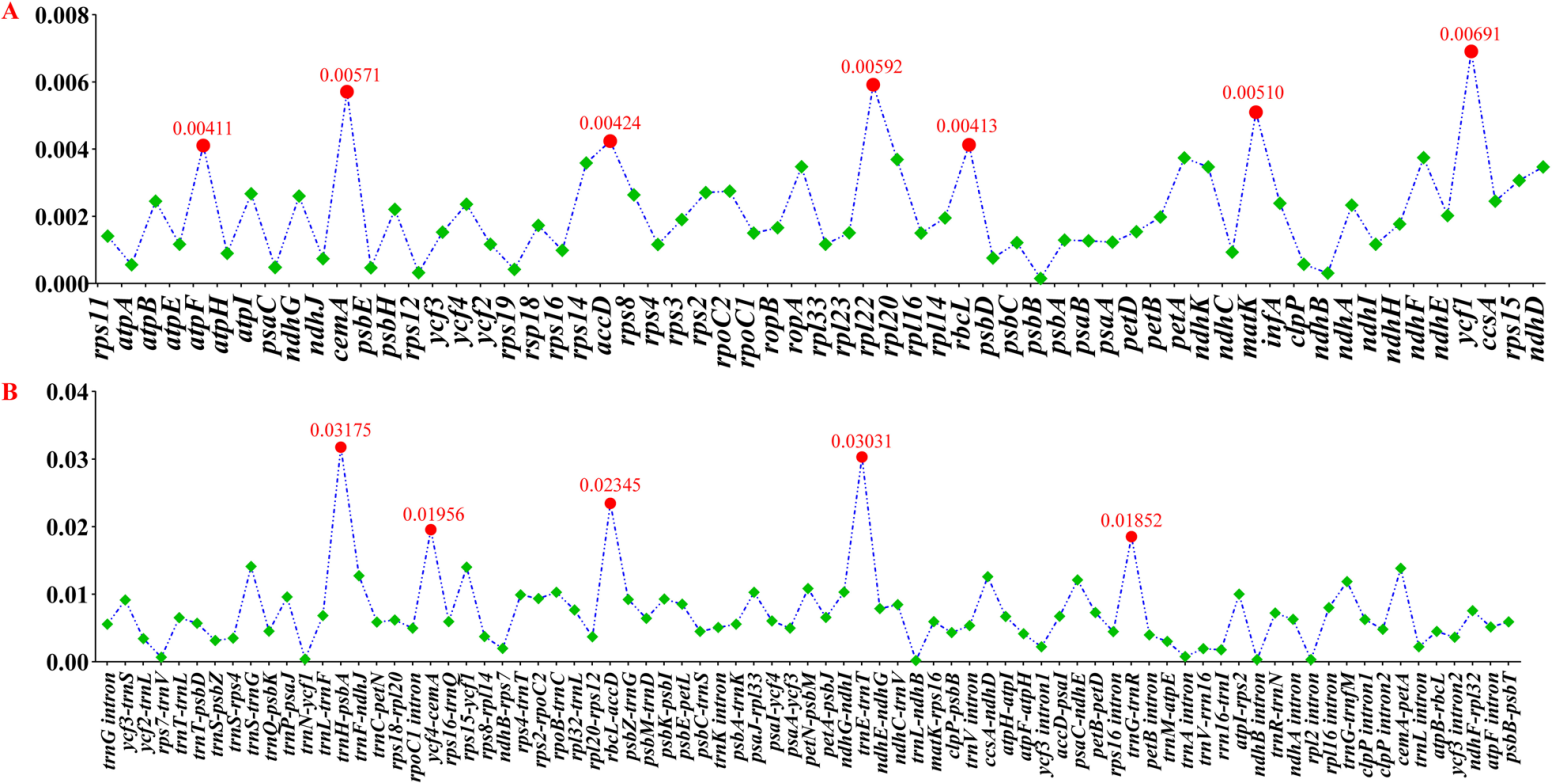
The nucleotide diversity (Pi) values among seventeen *Sanicula* plastomes. **A** protein coding regions; (**B**) non-coding and intron regions

High nucleotide diversity (Pi) value means that certain regions have large variation and can be used to design the potential DNA barcodes. In this study, we calculated Pi values of four regions among the *Sanicula* plastomes. The results showed that there were 146 sites, including 107 sites in the LSC region, 19 sites in the SSC region and 20 sites in the IRs regions (Table S4). The findings also indicated that the Pi value of non-coding region was remarkably higher than that of the coding region (Fig. 7). Based on the sequence divergence, twelve mutation hotspot regions were selected as candidate DNA barcodes, containing seven protein coding genes (*atp*F, *cem*A, *acc*D, *rpl*22, *rbc*L, *mat*K and *ycf*1) and five non-coding protein coding genes (*trn*H*-psb*A, *ycf*4*-cem*A, *rbc*L*-acc*D, *trn*E*-trn*T and *trn*G*-trn*R). The seven protein coding genes with the highest sequence variation had obviously higher values of 0.00411 (0.79%), 0.00571 (1.16%), 0.00424 (0.7%), 0.00592 (1.02%), 0.00413 (0.85%), 0.00510 (0.73%) and 0.00691 (1.47%), respectively (Fig. 7A, Table S4). Among them, six genes were located in the LSC region and one gene (*ycf*1 gene) was located at the SSC/IR junction. Five non-coding genes were considered as highly divergent hotspots with values of 0.03175 (7.07%), 0.01956 (0.99%), 0.02345 (2.81%), 0.03031 (1.99%) and 0.01852 (3%) (Fig. 7B, Table S4), all of which were located in the LSC region.

### Phylogeny analyses for genus *Sanicula*

**Fig. 8:**
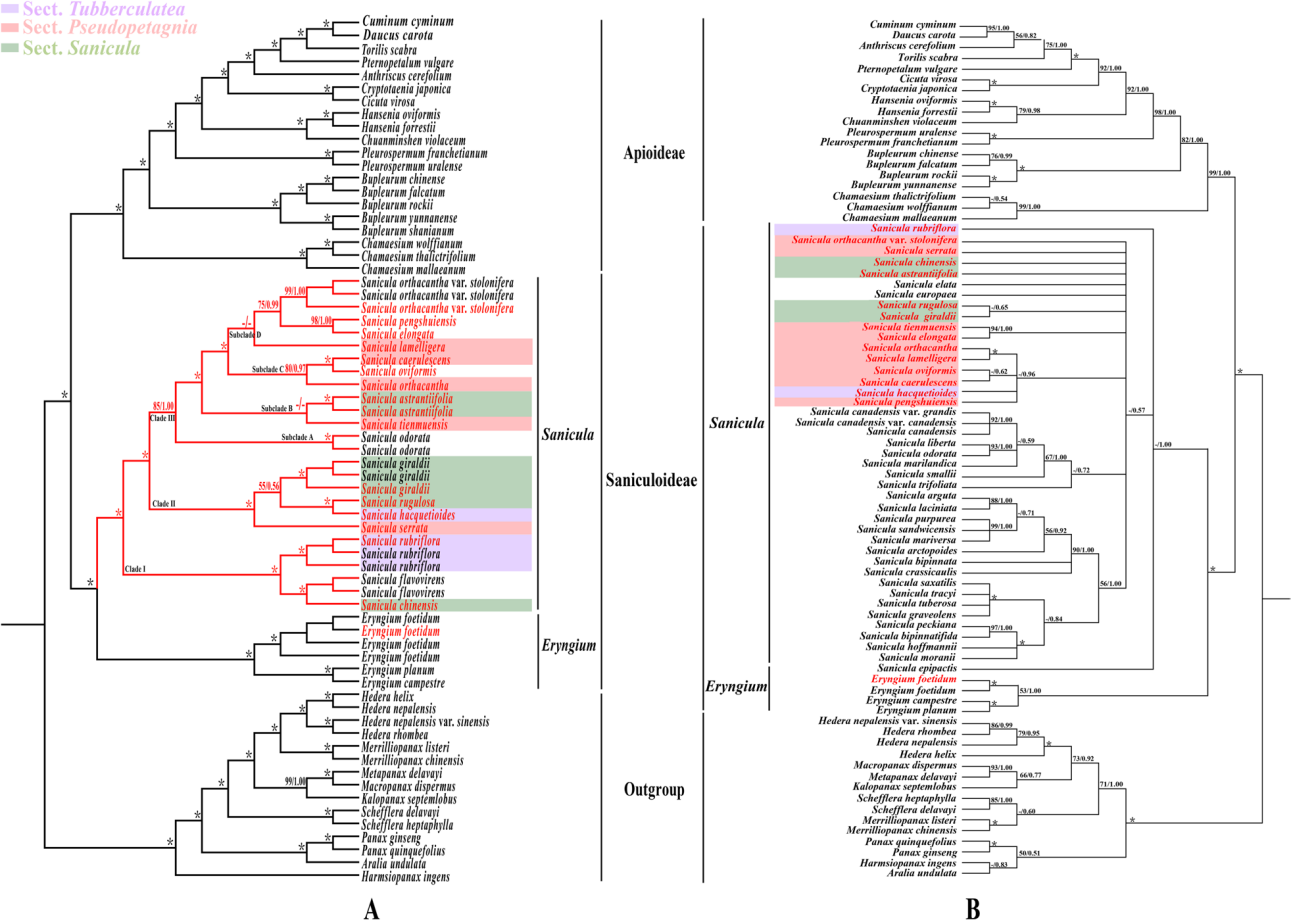
Phylogenetic trees constructed by maximum likelihood (ML) and Bayesian inference (BI). The bootstrap values (BS) of ML and posterior probabilities (PP) of BI are listed at each node. (*) represents the node with PP = 1.00/ BS = 100. – means the values < 0.50/50. Red words indicates the newly sequenced species. **A** CDS tree; **B**: ITS tree

The 79 shared CDSs of 67 complete plastomes and 80 ITS sequences were performed to reconstruct the phylogeny of *Sanicula* (Table S5). Although the tree topologies of the plastome data and ITS sequences were incongruent, both strongly suggested that the monophyly of Saniculoideae and Apioideae. In addition, sister groups between Saniculoideae and Apioideae were robustly supported. The *Sanicula* species involved in this study clustered together and the *Eryngium* species also clustered together (Fig. 8).

In the CDS-based phylogenetic tree, the results of the maximum likelihood (ML) and Bayesian inference (BI) analyses yielded a well-resolved topologies and the topologies were highly identical as expected (Fig. 8A). It was clearly observed that the sections of *Sanicula* involved in the current study were not respectively recovered as monophyletic group. Instead, seventeen members of *Sanicula* were obviously divided into three clades. *S. rubriflora* F. Schmidt ex Maxim. clustered with *S. flavovirens* and *S. chinensis*, belonging to Clade I with robust support (PP = 1.00, BS = 100). Clade II included four species (*S. serrata*, *S. rugulosa*, *S. hacquetioides*, and *S. giraldii* H. Wolff.). The remaining ten species (*S. odorata*, *S. astrantiifolia*, *S. tienmuensis*, *S. orthacantha*, *S. oviformi*s X.T. Liu & Z.Y. Liu., *S. caerulescens*, *S. elongata* K.T. Fu., *S. pengshuiensis*, *S. lamelligera*, and *S. orthacantha* var. s*tolonifera*) gathered together in Clade III, which could be divided into four subclades. It was noticed that Subclade A contained only one species: *S. odorata*. In Subclade B, *S. astrantiifolia* was sister to *S. tienmuensis* with weak supports (BS < 50, PP = 1.00). *S. orthacantha*, *S. oviformi*s and *S. caerulescens* formed a strong Subclade C (PP = 1.00, BS = 100), of which *S. oviformi*s and *S. caerulescens* clustered together and then resolved as sister to *S. orthacantha*. *S. elongata*, *S. pengshuiensis*, *S. lamelligera* and *S. orthacantha* var. s*tolonifera* belonged to Subclade D, of which *S. elongata* clustered with *S. pengshuiensis* with more closely relationship (Fig. 8A).

The topologies of ITS tree obtained from ML and BI analyses were also highly identical (Fig. 8B). Although weakly supported in the ITS analyses, both analyses also suggested that the sections of *Sanicula* were not monophyletic group. The phylogenetic position of most species were consistent with the CDS-based phylogenetic trees, but there were a little conflicts. For example: (1) *S. elongata* clustered with *S. pengshuiensis* in the CDS-based phylogenetic trees (BS = 98, PP = 1.00), while the former formed a clade with *S. tienmuensis* in the ITS phylogenetic trees (BS = 94, PP = 1.00); (2) *S. rugulosa* was sister to *S. hacquetioides* in the CDS-based phylogenetic trees (BS = 100, PP = 1.00), whereas the former resolved sister to *S. giraldii* with depressed support (BS < 50, PP = 0.65) in the ITS phylogenetic trees; (3) *S. orthacantha* formed a clade with *S. oviformis* and *S. caerulescens* in the CDS-based phylogenetic trees (BS = 80, PP = 0.97), while *S. orthacantha* gathered together with *S. lamelligera* in the ITS phylogenetic trees with robust support (BS = 100, PP = 1.00); (4) *S. tienmuensis* clustered with *S. astrantiifolia* in the CDS-based phylogenetic trees (BS < 50, PP < 0.5), whereas the former was sister to *S. elongata* with strong support (BS = 94, PP = 1.00) in the ITS analyses (Fig. 8B).

### Divergence time estimation

**Fig. 9:**
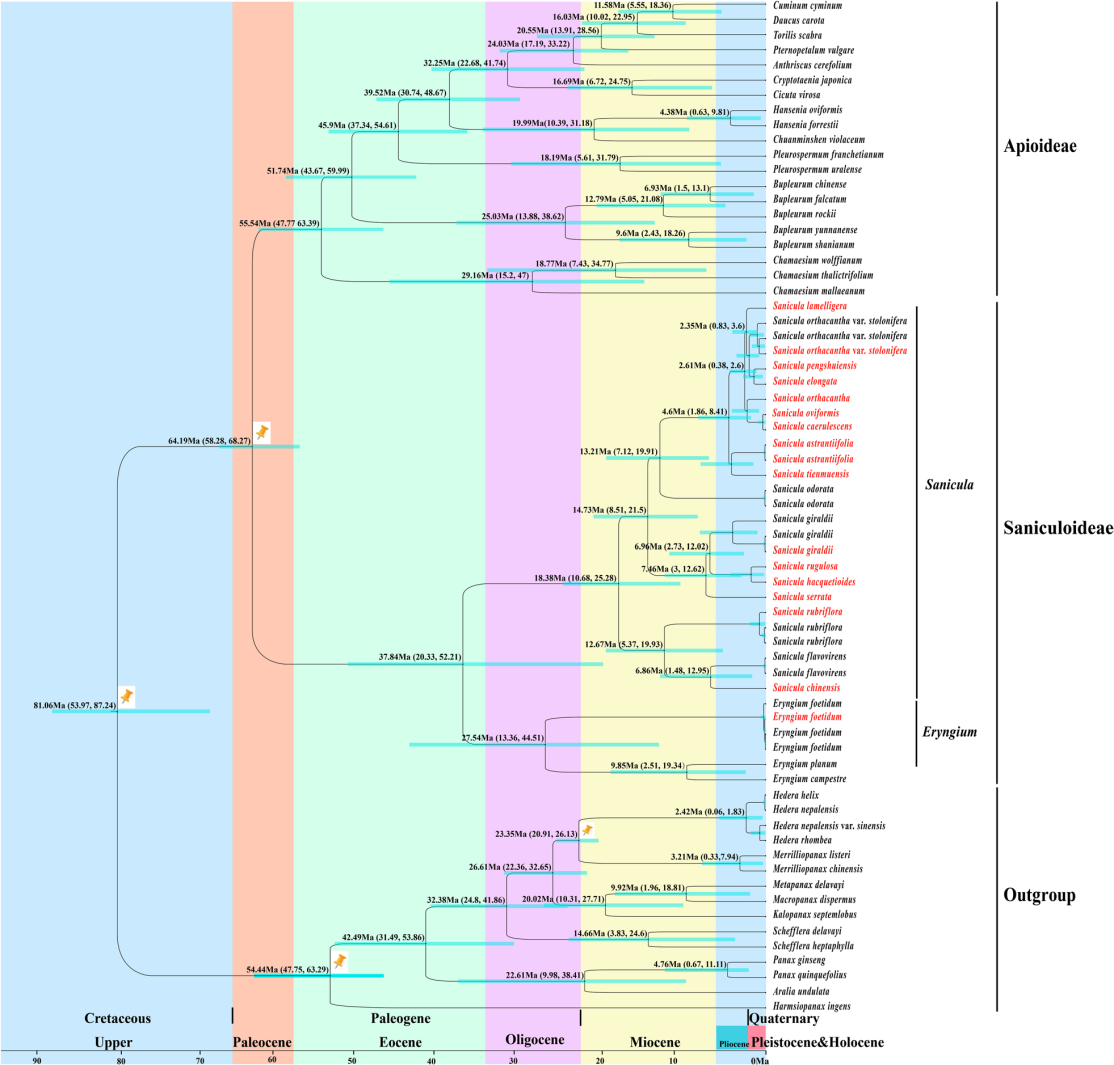
The divergence time estimation based on 79 shared genes. The maximum credibility tree from the divergence times estimated with BEAST. The 95% highest posterior density (HPD) estimates for each well-supported clade are represented by bars. Orange nails indicate the calibration points for the molecular dating

Based on four calibration points (one leaf macrofossil of Araliaceae and three calibration points constrained to a lognormal distribution), we estimated the divergence time of the genus *Sanicula*. Molecular dating results indicated that the family Apiaceae diverged from the family Araliaceae at 81.06 Ma (95% HPD: 53.97–87.24 Ma) in the Upper-Cretaceous. For the family Apiaceae, the divergence time between subfamily Apioideae and subfamily Saniculoideae was estimated to be 64.19 Ma (95% HPD: 58.28–68.27) when was from the Upper-Cretaceous to the late Paleocene period. Subsequently, the subfamily Apioideae diverged at around 55.54 Ma (95% HPD: 47.77–63.39 Ma) in the early Eocene period. Within the subfamily Saniculoideae, the divergence time between the genus *Sanicula* and *Eryngium* occurred at 37.84 Ma (95% HPD: 20.33–52.21 Ma) in the late Eocene period. In addition, the diversification of *Sanicula* were happened at 18.38 Ma (95% HPD: 10.68–25.28 Ma) in the early Miocene period (Fig. 9).

## Discussion

### Comparative analysis of *Sanicula* plastomes

Comparative analyses of plastome can provide valuable insights into understanding patterns of molecular evolution [44, 45]. In this study, a comprehensive comparison of seventeen *Sanicula* plastomes were carried out. All *Sanicula* plastomes had a typical quadripartite structure, consisting of one LSC region, one SSC region and a pair of IRs region (Fig. 1). Similarly, this structure was also detected in other plastomes of angiosperm plants [46–48]. Although gene loss is a common evolutionary event that frequently occurs in other genera of Apiaceae, such as the *ycf*15 gene was lost in genus *Peucedanum* [31] and the *trn*T-GGU gene was lost in genus *Peucedanum* and *Kitagawia* [31, 35], it was not discovered in genus *Sanicula*. In addition, the gene arrangement and GC content of the seventeen plastomes displayed high similarity. These findings demonstrated that the *Sanicula* plastomes were quite conserved. It is noteworthy that the GC content in the IR regions was relatively higher than that in the LSC and SSC regions (Table 1). The uneven distribution of GC content may be attributed to the higher GC content of the four rRNA genes (*rrn*16, *rrn*23, *rrn*4.5, and *rrn*5) in the IR regions, which is a common feature observed in other plants [49–51], as well as in Apiaceae [30–32].

IR contraction and expansion is a common phenomenon in the Apiaceae plastomes [20, 30–32, 35–40]. This phenomenon was also observed in *Sanicula* plastomes. Our study displayed that the *rps*19 gene had the length of 221 bp in the LSC region and 58 bp in the IRa region in sixteen plastomes, but in *S. flavovirens*, the entire *rps*19 gene was located in the LSC region. In these seventeen plastomes (except *S. flavovirens*), the *rpl*2 gene was 115–118 bp away from the IRb/LSC borders and the *trn*H gene was 2 bp away from the IRb/LSC borders, whereas in *S. flavovirens*, the *rpl*2 gene was only 32 bp away from the IRb/LSC borders, and the *trn*H gene was 87 bp away from the IRb/LSC borders, which resulted in shorter plastome length of *S. flavovirens* than in the other sixteen *Sanicula* species. Therefore, we supported the hypothesis that the genome size variation was caused by IR contraction and expansion. Furthermore, we also examined the inverted repeats types described in the Apiaceae [20, 30–32, 35–40] and found that the situation observed in the genus *Sanicula* was also detected in the other genera of Apiaceae, such as in *Hansenia* Turcz., *Haplosphaera* Hand.-Mazz., *Sinodielsia* H. Wolff [37].

### Plastome evolution of *Sanicula*

Codons provide a link between nucleic acids and proteins, conveying genetic information [39]. The patterns of codon usage show basic characteristics of molecular evolution [52]. In species, codon usage is not random, as some synonymous codons are used more frequently than others, a phenomenon known as codon usage bias (CUB) [53]. CUB is considered to be a driver of gene evolution and it provides a great deal of information for understanding molecular evolution [54]. It exists in all species’s genomes and is influenced by many factors, such as mutation pressure, natural selection, gene composition and gene length [55]. In the present study, all codons with RSCU > 1.00 were strongly biased towards A/U at the third codon position in seventeen *Sanicula* plastomes, which was consistent with the observation in most angiosperms [56], as well as in Apiaceae [30–32, 35–39]. Perhaps the high AT content in plastome is the major reason for the bias towards A/U for codons ending in A/U [39]. Leucine was the most abundant amino acid and was encoded by the highest number of codons, while cysteine was the rarest amino acid, as in most Apiaceae species [30–32].

Repeat sequences can cause gene insertion, deletion, substitution and duplication, which can lead to the generation of divergent regions in genome rearrangements. Therefore, these repeats can provide crucial information for phylogenetic and population studies [57]. Among the seventeen *Sanicula* plastomes, palindromic repeats were the majority of the four types of repeats, which have also been reported in the genus *Ligusticum* [30] and *Cnidium* [39].

SSRs are shorter tandem or microsatellite repeat sequences with repeat units of 1–6 bp, which are widely distribute in different regions of the plastome [58]. Due to a large amount of variation within the species, SSRs have been extensively used as valuable molecular markers for species authentication, population evolutionary analyses, phylogenetic relationship analyses, plant taxonomy, geography of species, genetic diversity and population structure studies [59]. Our research identified 979 SSRs in seventeen *Sanicula* plastomes. Mononucleotide were the most abundant SSRs, followed by dinucleotide, tetranucleotide, trinucleotide, hexanucleotide and pentanucleotide repeats. In addition, most of the SSRs contained A/T motifs, resulting in the AT richness of the seventeen *Sanicula* plastome. The similar pattern has also been observed in other Apiaceae plants [32]. In particular, the longest SSR was hexanucleotide (ACATAT/ATATGT) and it can serve as genetic markers in population genetics and phylogeography studies of *Sanicula* in the future.

#### Candidate DNA barcodes

With the rapid development of sequencing technologies and DNA barcoding, variable loci (e.g., *mat*K, *rbc*L and *trn*H*-psb*A) have been recognized as universal DNA barcodes that were widely used for species identification, resource management, phylogenetic analyses and species evolution research in land plants [31, 36, 60, 61]. Although universal DNA barcoding have successfully distinguished many species, they have failed for some taxonomically notorious taxa and have encountered weak solutions [62]. Therefore, it is urgently needed to develop more effective variable loci as candidate DNA barcodes to solve the dilemma. Since plastome contain large-scale differentiation hotspots, thus it has the strong potential for screening DNA barcodes [63, 64]. In the current study, we investigated that the sequence variability in the non-coding region was higher than in the coding region, which was also detected in other genera of Apiaceae [31, 32]. Moreover, we identified five non-coding protein coding genes (*trn*H*-psb*A, *ycf*4*-cem*A, *rbc*L*-acc*D, *trn*E*-trn*T and *trn*G*-trn*R) and seven protein coding genes (*atp*F, *cem*A, *acc*D, *rpl*22, *rbc*L, *mat*K and *ycf*1) that could be used as candidate DNA barcodes for *Sanicula* species identification. Of these twelve DNA barcodes, except for three universal DNA barcodes (*rbc*L, *mat*K and *trn*H*-psb*A) and two promising DNA barcodes (*acc*D and *ycf*1) in some plants [65], the remaining seven high variable regions (*atp*F, *cem*A, *rpl*22, *ycf*4*-cem*A, *rbc*L*-acc*D, *trn*E*-trn*T and *trn*G*-trn*R) were different from those of other species [31, 66]. Therefore, they could be regarded as specific DNA barcodes to distinguish *Sancilua* species. These findings offered a valuable reference for further developing DNA barcodes for *Sanicula*.

### Phylogeny inference and taxonomic implication of *Sanicula*

The plastome is uniparental inheritance, lacks recombination, and has highly variable loci; thus, it has been widely used in phylogenetic studies of angiosperms, especially at the low taxonomic levels of Apiaceae [20, 30–32, 35–40, 66, 67]. In the present study, the plastome data generated a well-resolved phylogenetic tree for *Sanicula*. The supports and resolutions of the plastome-based tree were significantly improved compared to those of the ITS-based tree constructed in the current and previous studies [5, 13, 15, 17].

The incongruences between ITS-based and plastome-based phylogenies are a common phenomenon in Apiaceae [31, 33, 35, 36, 67], and our results are no exception. These incongruences are mainly caused by hybridization/introgression, incomplete lineage sorting (ILS) and chloroplast capture events [20], as well as the maternal inheritance of plastid and biparental inheritance of ITS, which may also be an important factor [30].

Although topological incongruence existed between the plastome data and the ITS sequences, both successfully recognized all *Sanicula* species involved in the current study clustered together, which was consistent with several previous molecular phylogenetic analyses [5, 7, 12, 15, 17–19, 42]. Meanwhile, both topologies supported the sections within the genus were not being monophyletic group and the current classification may be inappropriate. Therefore, we considered that the current section-level classification system of *Sanicula* needs to be improved and revised. Although the current study did not provide a new taxonomic treatment of *Sanicula* taxa due to lack of insufficient sampling or the low supports and resolutions, our study laid the foundations and served as a framework for future taxonomic studies of *Sanicula*.

In addition, we clarified the taxonomic relationships of the four species. In the present study, both analyses indicated that *S. orthacantha* var. *stolonifera* was relatively far from *S. orthacantha.* Their notably different morphological characters of the two taxa further supported the phylogenetic results. For example, the rhizomes features (short, tuberlike, woody, oblique rootstock bearing elongated, fibrous roots, occasionally having fleshy stoloniferous) and narrow-linear, acute calyx teeth were observed in *S. orthacantha*, while slender, elongate and lignified nodes stoloniferous rhizomes and ovate calyx teeth were found in *S. orthacantha* var. *stolonifera* [13]. Thereby, *S. orthacantha* var. *stolonifera* should be regarded as an independent species rather than a variety of *S. orthacantha* based on the molecular and morphological evidence. A published study has treated *S. pengshuiensis* as a synonymy of *S. lamelligera* [14]. Although the relationship between the two species was unsolved in ITS-based tree, the plastome-based tree robustly supported that *S. pengshuiensis* clustered with *S. elongata*. The morphological characteristics of them also supported the above phylogenetic results, such as inflorescence cymose branched, peduncles elongate was detected in *S. pengshuiensis*, while inflorescence 2–4-dichotomously or trichotomously branched, sometimes subcorymbose was found in *S. lamelligera* [13]. Hence, we suggested that *S. pengshuiensis* should be regarded as an independent species rather than a synonymy of *S. lamelligera*.

#### Estimation divergence time

Our analysis based on date estimation revealed that the divergence between the family Araliaceae and Apiaceae occurred during the Upper-Cretaceous period, with an approximate median age of 81.06 Ma (95% HPD: 53.97–87.24 Ma). This time was roughly consistent with the findings of Xie et al., who also used plastomes to estimate the divergence time of Apiales [40], but earlier than some studies (between 60 and 72 Ma [20, 68]. As for the family Apiaceae, the subfamilies Apioideae and Saniculoideae diverged at the beginning of the Paleogene, approximately 64.19 Ma (95% HPD: 58.28–68.27 Ma). The time was slightly earlier than other previous studies [20, 68, 69], but aligned closely with findings inferred from *trn*D*-trn*T *+ rpl*16 intron, with a deviation of 65.78 Ma (95% HPD: 58.21–74.31 Ma) [70]. The diversification of the subfamily Apioideae was estimated to have occurred during the early Eocene period 55.54 Ma. This time was earlier than that suggested by previous studies conducted by Wen et al. (43.45 Ma and 49.78 Ma) [20, 68]. On the other hand, in subfamily Saniculoideae, the genus *Sanicula* and *Eryngium* genera took place during the late Eocene period, precisely 37.84 Ma years ago (95% HPD: 20.33–52.21 Ma). This estimation was comparatively earlier than the previous records [20]. The potential discrepancy between our study and earlier previous researches may be caused by the differences in sampling and fossil calibrations used [40, 71]. Our results were considered more accurate and credible because the study contained a larger sample of *Sanicula* taxa and a broader range of calibration points, which were frequently employed in other evolutionary analyses [18, 40, 71, 72]. Therefore, we obtained a more precise divergence times.

Within subfamily Saniculoideae, the origin of genus *Sanicula* was estimated to have occurred during the late Eocene (37.84 Ma). Previous studies have reported that the initial uplift of the Qinghai-Tibetan Plateau (QTP) occurred at 45–35 Ma [71]. The QTP, which extends to the Hengduan Mountains (HDM) located on the southeastern edge of the current QTP [73], encompasses the most extensive range. Thus, the monolithic uplift of QTP led to the uplift of HDM, and some studies have suggested that the early uplift of the southern part of the HDM can be traced back to the period of late Paleocene or even the Eocene [74]. The uplift of the HDM resulted in the formation of a highly complex topography, giving rise to the creation of distinct and isolated “sky islands” [75, 76]. The “sky islands” have played a vital role in plant origin, speciation and evolution throughout the HDM and neighbouring regions, which has resulted in the emergence and diversification of various lineages, including *Plethodon ouachitae*, *Saussurea* and *Cupressus* L [77–80]. Therefore, we hypothesized that the uplift of the HDM may also have contributed to the origin of *Sanicula* species. The diversification of *Sanicula* occurred at 18.38 Ma, during the early Miocene. This period coincided with significant changes in East Asian monsoon intensity due to global temperature fluctuations [81], which led to substantial transformations in numerous biological communities in East Asia. This environmental shifts potentially facilitated the diversification of many species, such as *Primulina*, *Lepisorus* and *Begonia* [82–84]. Therefore, we assumed that the intensification of the East Asian monsoon might also acted as an indispensable role in promoting the diversification of *Sanicula.* Moreover, the uplift of HDM also likely influenced the diversification of this genus. The HDM experienced extensive uplift caused by the collision between the Indian plate and Eurasia, occurring after the Miocene and reaching its peak elevation before the Late Pliocene [85, 86]. This geological event triggered a series of topographical and climatic changes. The altered topography provided diverse habitats that facilitated species differentiation, as observed in the cases of *Caragana* and *Lilium* [87, 88]. Therefore, we supposed that the diversification of *Sanicula* species was closely related to the prevalence of the East Asian monsoon and the uplift of HDM events.

## Conclusion

In this study, we successfully sequenced and assembled the complete plastomes of fifteen *Sanicula* species. Together with two previously reported plastomes, we performed a comprehensively comparative analyses. The results revealed that the genome structure, gene number, GC content, gene rearrangement and codon usage of seventeen *Sanicula* plastomes were highly conserved. Nevertheless, twelve highly variable regions were still selected as potentially strong DNA barcodes, which exhibited superior efficacy in species identification and phylogenetic relationship construction. Phylogenetic analyses indicated that the *Sanicula* species involved in this study clustered together. However, the existing section classification system was deemed non-natural. The incongruences between ITS-based and plastome-based phylogenies were also observed in the genus. These discrepancies could largely be attributed to the events of hybridization/introgression, incomplete lineage sorting (ILS) and chloroplast capture, and the maternal inheritance of plastids, while the biparental inheritance of ITS. Molecular clock analysis indicated that the origin of genus *Sanicula* occurred during the late Eocene, which was significantly correlated with the uplift of the HDM. And the diversification of the genus occurred in the early Miocene, which was largely influenced by the prevalence of the East Asian monsoon and the uplift of the HDM. In conclusion, our study holds substantial significance and value for the investigation of the taxonomy, phylogeny, and evolution of the *Sanicula* genus.

## Methods

### Plant material and DNA extraction

The samples of fifteen *Sanicula* taxa were collected from the wild and then the fresh basal young leaves were immediately dried and stored with silica gel for further DNA extraction. The formal identification of these taxa was carried out by Professor Xingjin He (Sichuan University). Vouchers were stored in the herbarium of Sichuan University (Chengdu, China) under the deposition number listed in Table S6. Total DNA of newly collected samples was extracted from silica gel-dried fresh leaf tissues using the modified CTAB method [89]. We also newly sequenced fifteen *Sanicula* ITS sequences and submitted them to NCBI (accession numbers: OQ651137-OQ651152) ( Table S5). To re-evaluate the taxonomic system of the genus more objectively, we also downloaded the plastomes and ITS sequences of this genus that were currently available on the NCBI.

### Genome sequencing, assembly and annotation

The quality and quantity of DNA were tested using 1% agarose gel electrophoresis, and high-quality DNA was sequenced on an Illumina HiSeq2500 platform from Novogene (Beijing, China) according to the standard Illumina sequencing protocols [90]. Paired-end 150 reads were generated from libraries with an insert size of 300 bp. The raw data were filtered by software fastP v0.15.0 to obtain high-quality reads, with -n 10 and -q 15 [91]. For plastome assembly, we employed two strategies. First, we used NOVOPlasty v2.7.2 [92] to assemble complete plastomes, with the default parameters and *rbc*L sequence extracted from the plastome of *S. lamelligera* (MT561031) as the seed. To validate the accuracy of plastome assembly, GetOrganelle pipeline [93] was also used to assemble the plastomes, using the plastome sequence of *S. lamelligera* (GenBank accession: MT561031) as reference. And the same assembled results were obtained by both methods. The assembled plastomes were initially annotated with the program DOGMA [94]. Then, the start and stop codons and intron positions were checked and manually corrected when necessary in Geneious v9.0.2 [95] and the plastomes of a given species recovered by different assembly strategies were also compared using Geneious v9.0.2 [95]. The circular plastome map was depicted by online program CHLOROPLOT (https://irscope.shinyapps.io/Chloroplot/) [96] and the newly sequenced and assembled seventeen complete plastomes were submitted into NCBI (accession numbers: OQ612639-OQ612643 and OQ626817-OQ626828) (Table S5).

### Codon usage bias

The protein-coding genes (PCGs) were extracted from seventeen *Sanicula* plastomes for codon analysis. All overlapping genes were removed and the remaining 79 PCGs for each species. Since shorter genes may bias the codon usage estimation [97] and to avoid sampling bias [98], 79 PCGs with CDSs length shorter than 300 bp were excluded from this study, and 53 CDSs were finally screened. Relative synonymous codon usage (RSCU) is the value of the observed and expected frequency of a codon encoding a specific amino acid [99]. The RSCU value is divided into three ranges by the value 1.00. Among them, the RSCU value > 1.00, = 1.00, and < 1.00 represents positive codon usage bias, no bias, and negative codon usage bias, respectively. Especially, codons with RSCU values greater than 1.6 and less than 0.6 are regarded as “over-represented” and “under-represented” codons, respectively [100]. The RSCU values were calculated using the CodonW v1.4.2 program [101] and the RSCU heatmap of the seventeen plastomes was emerged by GraphPad Prism 7 [102].

### Repeat sequence and simple sequence repeats (SSRs) analysis

The four repetitive sequences: Forward (F), Reverse (R), Palindromic (P) and Complementary (C) repeats of the seventeen *Sanicula* plastomes were detected by the online REPuter program [103]. The parameters were set with maximum computed repeats > 90%, minimal repeat size ≥ 30 bp, and a hamming distance = 3. All overlapping repeat sequences were removed. Simple sequence repeats (SSRs) were then checked using the MISA Perl script [104]. The minimum number of repeat units parameter was set to 10, 5, 4, 3, 3, and 3 for mono-, di-, tri-, tetra-, penta-, and hexanucleotides, respectively.

### Comparative plastome of *Sanicula*

We compared the boundaries between inverted repeat and single-copy (IR/SC) among the seventeen plastomes in Geneious v9.0.2 [95]. The junction regions of LSC/IRb/SSC/IRa were visualized by IRscope [105] after manually adjusted. Then, the DNA rearrangements among seventeen *Sanicula* plastomes were detected by using Mauve Alignment [106] implemented in Geneious v9.0.2 [95], with other parameters set as the default values. Furthermore, we also investigated the sequence divergence of *Sanicula* plastomes using mVISTA viewer [107], setting *S. astrantiifolia* as the reference.

### Identification of high divergence hotspots

The protein coding regions, non-coding regions and intron regions of the seventeen plastomes were extracted in Geneious v9.0.2 [95] to identify mutation hotspot regions. We then aligned the sequences using MAFFT v7.221 [108] and the sequences were manually corrected with BioEdit software [109] and Geneious v9.0.2 [95]. And the alignment with less than 200 bp in length were discarded, mainly because the relatively short and insufficient sequence can yield the variation [110]. Finally, we evaluated nucleotide diversity (Pi) [111] using DnaSP version 5.0 software [112] to further investigate the molecular evolution of the *Sanicula* plastome.

### Phylogenetic analysis

To illustrate the phylogenetic relationship of genus *Sanicula*, 67 plastomes and 80 ITS sequences were used to reconstruct the phylogenetic tree (Table S5). Among them, fifteen species of family Araliaceae were chosen as outgroup referred to the phylogenetic result of previous study [40]. The 80 ITS sequences were straightway aligned with MAFFT v7.221 [107] to gain the matrix. For plastome data, 79 commonly shared CDSs of 67 species were manually extracted in Geneious v9.0.2 [95] and aligned with MAFFT v7.221 [107]. Then the alignments were concatenated as a super matrix with PhyloSuite v1.2.2 [113]. Two matrixes were subjected to Maximum-Likelihood analysis (ML) and Bayesian Inference (BI). In detail, for the ML analyses, RAxML v8.2.8 [114] with GTRGAMMA model was performed to reconstruct the phylogenetic tree, and 1000 replicates was suggested to estimate the support value (BS) for each node according to the RAxML manual. Bayesian inference was performed by MrBayes v3.2.7 [115] and the best-fit nucleotide substitution model (GTR + I + G) for matrix of concatenated plastid protein-coding and (SYM + I + G) for ITS dataset were determine by Modeltest v3.7 [116] under the Akaike Information Criteria (AIC) [117]. The Markov chain Monte Carlo (MCMC) algorithm was run for 5,000,000 generations (sampling every 100 generations) with two runs and four chains (three heated chains and one cold chain). The running finished until the average standard deviation of split frequencies was below 0.01. An initial 25% of sampled trees were discarded, and the remainder were retained to generate the consensus tree and calculate posterior probabilities (PP). The results of ML and BI phylogenetic analyses were visualized in FigTree v1.4.2 [118].

### Divergence time estimation

Bayesian relaxed clock analysis in program Bayesian Evolutionary Analysis Sampling Trees (BEAST v1.10.4) [119] was performed to estimate the divergence time of genus *Sanicula*. In the preliminary experiment, we analyzed random trees because the starting tree could not converge after two runs, but the ML phylogenetic tree was almost fully supported. Thus, the ML tree inferred from matrix of concatenated plastid protein-coding was used to fix topology. One leaf macrofossil of Araliaceae and three calibration points constrained to a lognormal distribution were used to constrain the phylogenetic tree. (i) the stem node of *Hedera* L. was constrained to a minimum age of 23.0 Ma based on leaf macrofossil of *Hedera* sp [120]. ; (ii) based on the fossilized leaves of Araliaceae, the stem node of *Harmsiopanax ingens* Philipson. was set as 49.28–72.9 Ma age [121]; (iii) according to the study of Wen et al., the root point was set set to 68.88 Ma (95% HPD: 53.97–87.24 Ma) [20]; (iv) referring to the previous research of the evolutionary timescale, 54.17 Ma (95% HPD: 45.55–66.44) was implemented as a minimum age of subfamily Apioideae and Saniculoideae [20]. BEAUti was used to set criteria for analysis under the uncorrelated relaxed molecular clock model, with a Yule tree prior and the best-fit nucleotide substitution model (GTR + I + G) detected by Modeltest v3.7. Two independent replications of MCMC simulations were run for 2 × 10^9^ generations with sampling every 2000 generations, with the first 20% trees being discarded as burn-in and the convergence of remaining runs was assessed through Tracer v1.7.1 [122] to examine the effective sample size (ESS) of all parameters not less than 200. The maximum clade credibility tree with median ages and 95% highest posterior density (HPD) intervals were produced with TreeAnotator v2.1.2 [123] and the final result was exhibited in FigTree v1.4.2 [118].

### Supplementary Information

**Additional file 1: Table S1. **List of unique genes identified in plastomes of *Sanicula.*

**Additional file 2: Table S2. **Codon usage and relative synonymous codon usage (RSCU) values of 53 protein-coding genes of seventeen *Sanicula* plastomes.

**Additional file 3: Table S3.** Repeat sequence and simple sequence repeats in seventeen *Sanicula* plastomes.

**Additional file 4: Table S4.** Nucleotide diversity (Pi) of coding and non-coding regions.

**Additional file 5: Table S5.** The plastomes data included in phylogenetic analyses with GenBank accession.

**Additional file 6: Table S6.** The sample information of seventeen *Sanicula* in this study

## Data Availability

The datasets generated and/or analyzed during the current study are available in the NCBI repository, https://www.ncbi.nlm.nih.gov/. Accession numbers: OQ612639-OQ612643, OQ626817- OQ626828 and OQ651137-OQ651152.

## Abbreviations

CTAB: Cetyl trimethylammonium bromide
PCR: Polymerase chain reaction
ITS: Internal transcribed spacer
CDS: Protein-coding sequences
bp: Base pair
BI: Bayesian inference
PP: Posterior probability
BS: Branch support
AIC: Akaike information criterion
ML: Maximum Likelihood
MCMC: Markov chain Monte Carlo
IR: Inverted repeat
LSC: Large single copy
SSC: Single copy
SSR: Simple sequence repeat
RSCU: Relative synonymous codon usage
Pi: Nucleotide diversity
rRNA: Ribosomal RNA
tRNA: Transfer RNA
