# Supplementary material for: Phylogeny and diversification of genus Sanicula L. (Apiaceae): novel insights from plastid phylogenomic analyses
Source: BMC Plant Biol. 2024 Jan 24;24:70. doi: 10.1186/s12870-024-04750-0 (PMC10807117; doi:10.1186/s12870-024-04750-0)
Supplement: Supplementary file 6 — Additional file 6: Table S6. The sample information of seventeen Sanicula in this study [file 12870_2024_4750_MOESM6_ESM.docx]

Table S6 The sample information of seventeen *Sanicula* in this study

| Taxon | Locality | Deposition numbers | Herbarium information |
| --- | --- | --- | --- |
| *Sanicula astrantiifolia* H. Wolff ex Kretschmer | Lijiang,Yunnan,China | SBN2022010 | Sichuan University |
| *Sanicula chinensis* Bunge | Huyi,Shanxi,China | SBN2022015 | Sichuan University |
| *Sanicula caerulescens* Franch | Nanchuan, Chongqing,China | SBN2022014 | Sichuan University |
| *Sanicula elongata* K.T. Fu | Zhouzhi,Shanxi,China | SBN2022013 | Sichuan University |
| *Sanicula giraldii* | Shouyang,Shanxi,China | SBN2022012 | Sichuan University |
| *Sanicula hacquetioides* Franch | Diqing,Yunnan,China | SBN2022011 | Sichuan University |
| *Sanicula lamelligera* Hance | Guilin,Guangxi,China | SBN202208 | Sichuan University |
| *Sanicula orthacantha* S. Moore | Chengdu,Sichuan,China | SBN202207 | Sichuan University |
| *Sanicula orthacantha* var. *stolonifera* Shan et S.L.Liou | Leshan,Sichuan,China | SBN202201 | Sichuan University |
| *Sanicula oviformi*s X.T. Liu & Z.Y. Liu | Nanchuan,Chongqing,China | SBN202206 | Sichuan University |
| *Sanicula pengshuiensis* M.L. Sheh & Z.Y. Liu | Shizhu,Chongqing,China | SBN202205 | Sichuan University |
| *Sanicula rubriflora* F. Schmidt ex Maxim | Haerbin, Heilongjiang,China | SBN202204 | Sichuan University |
| *Sanicula rugulosa* Diels | Yaan,Sichuan,China | SBN202203 | Sichuan University |
| *Sanicula serrata* H. Wolff | Kangding,Sichuan,China | SBN202202 | Sichuan University |
| *Sanicula tienmuensis* Shan & Constance | Tianmu,Zhejiang,China | SBN202209 | Sichuan University |
| *Sanicula flavovirens* Z.H.Chen, D.D. Ma et W. Y. Xie | / | / | / |
| *Sanicula odorata* (Raf.) Pryer & Phillippe | / | / | / |
